# Comparison of Saccadic Vector Optokinetic Perimetry and Standard Automated Perimetry in Glaucoma. Part I: Threshold Values and Repeatability

**DOI:** 10.1167/tvst.6.5.3

**Published:** 2017-09-06

**Authors:** Ian C. Murray, Antonios Perperidis, Lorraine A. Cameron, Alice D. McTrusty, Harry M. Brash, Andrew J. Tatham, Pankaj K. Agarwal, Brian W. Fleck, Robert A. Minns

**Affiliations:** 1 University of Edinburgh, Edinburgh, Scotland, United Kingdom; 2 Heriot Watt University, Edinburgh, Scotland, United Kingdom; 3 Glasgow Caledonian University, Glasgow, Scotland, United Kingdom; 4 Princess Alexandra Eye Pavilion, Edinburgh, Scotland, United Kingdom; 5 Royal Hospital for Sick Children, Edinburgh, Scotland, United Kingdom

**Keywords:** SVOP, eye movement perimetry, visual field, saccadic eye movements

## Abstract

**Purpose:**

We evaluated threshold saccadic vector optokinetic perimetry (SVOP) and compared results to standard automated perimetry (SAP).

**Methods:**

A cross-sectional study was done including 162 subjects (103 with glaucoma and 59 healthy subjects) recruited at a university hospital. All subjects underwent SAP and threshold SVOP. SVOP uses an eye tracker to monitor eye movement responses to stimuli and determines if stimuli have been perceived based on the vector of the gaze response. The test pattern used was equivalent to SAP 24-2 and stimuli were presented at Goldmann III. Average and pointwise sensitivity values obtained from both tests were compared using Pearson's correlation coefficient. Two versions of SVOP were evaluated.

**Results:**

A total of 124 tests were performed with SAP and SVOP version 2. There was excellent agreement between mean threshold values obtained using SVOP and SAP (*r* = 0.95, *P* < 0.001). Excluding the blind spot, correlation between SVOP and SAP individual test point sensitivity ranged from 0.61 to 0.90, with 48 of 54 (89%) test points > 0.70. Overall SVOP showed good repeatability with a Pearson correlation of 0.88. The repeatability on a point-by-point basis ranged from 0.66 to 0.98, with 45 of 54 points (83%) > 0.80. Repeatability of SAP was 0.87, ranging from 0.69 to 0.96, with 47 of 54 (87%) points > 0.80.

**Conclusion:**

Eye-tracking perimetry is repeatable and compares well with the current gold standard of SAP. The technique has advantages over conventional perimetry and could be useful for evaluating glaucomatous visual field loss, particularly in patients who may struggle with conventional perimetry.

**Translational Relevance:**

Suprathreshold SVOP already is in the field. To our knowledge, this is the first report of threshold SVOP and provides a benchmark for future iterations.

## Introduction

The use of a static white stimulus on a white background (white-on-white) has become the accepted standard for investigation of visual function in patients with glaucoma. White-on-white automated suprathreshold tests often are used for screening purposes,^1^ with threshold tests (standard automated perimetry, SAP) used to aid glaucoma diagnosis and quantify disease progression.^2^ Evaluating differential light sensitivity using threshold testing is an essential part of glaucoma management; however, full threshold testing is time-consuming, which can have an adverse effect on reliability. Although newer testing strategies, such as the Swedish Interactive Threshold Algorithm (SITA),^3,4^ have improved efficiency, many patients still find SAP difficult.^5,6^ The requirement to maintain fixation on a central target, and then signal perception of a peripheral stimulus, without altering fixation, can be challenging. In addition, patients are required to maintain a fixed head position, which may be problematic. All of these factors can lead to longer test times and contribute to test–retest variability and low test reliability.^7,8^

Saccadic vector optokinetic perimetry (SVOP) is a technique originally developed to enable suprathreshold visual field assessment in children unable to perform conventional forms of perimetry.^9^ SVOP uses eye tracking to assess the visual field by automated real-time assessment of natural eye movement responses to stimuli, and has no postural constraints.^9,10^ Different iterations of the technique have now been evaluated in children and adults.^11,12^

We have developed a version of SVOP for threshold testing. The purpose of this study was to introduce threshold SVOP and compare SVOP threshold values to threshold values obtained from SAP in patients with glaucoma and normal subjects. A second part to this study is continued in a companion paper, which compares visual field patterns obtained by threshold SVOP and SAP, and evaluates patients' perceptions of threshold SVOP.

## Materials and Methods

### Subjects

This cross-sectional study included 103 patients with glaucoma (age range, 42–91 years; mean age, 70.6 ± 8.79 years) and 59 healthy subjects (age range, 50–81 years; mean age, 65.7 ± 5.8 years). Patients with glaucoma were recruited from a nonconsecutive series of patients attending the glaucoma clinic at the Princess Alexandra Eye Pavilion, Edinburgh. Healthy subjects were required to have no previous history of glaucoma or visual field defect and no neurological conditions that might affect the visual field. All subjects provided written informed consent. The study adhered to the tenets of the Declaration of Helsinki and was approved by the South-East Scotland Research Ethics Committee, NHS Lothian.

All patients attending the glaucoma clinic had undergone best-corrected visual acuity assessment, slit-lamp biomicroscopy, intraocular pressure measurement, pachymetry, gonioscopy, and dilated funduscopy. Glaucoma was diagnosed by a glaucoma specialist, based on the presence of typical glaucomatous changes in optic disc morphology and the presence of a glaucomatous visual field defect on SAP, using the Humphrey visual field analyzer (HFA; Carl Zeiss Meditec, Dublin, CA) SITA Fast 24-2 test. Patients with strabismus or a history of eye movement disorders were excluded. All patients had a best corrected visual acuity of better than 0.3 logMAR. In the healthy group, eyes with a visual acuity worse than 0.15 logMAR were excluded.

## Test Methods

Each subject underwent SAP (24-2 SITA fast) and threshold SVOP on both eyes, with the order of testing randomized. A smaller cohort of subjects underwent repeat testing in one eye to evaluate repeatability. For glaucoma patients, repeat testing was performed on the eye with the more advanced SAP visual field loss. For healthy subjects, one eye was selected randomly for repeat testing. Testing was performed during a single session on the same day and breaks offered between tests should they be required. The SVOP and SAP tests used are described in more detail in the following sections.

### SVOP Test

**Figure 1:**
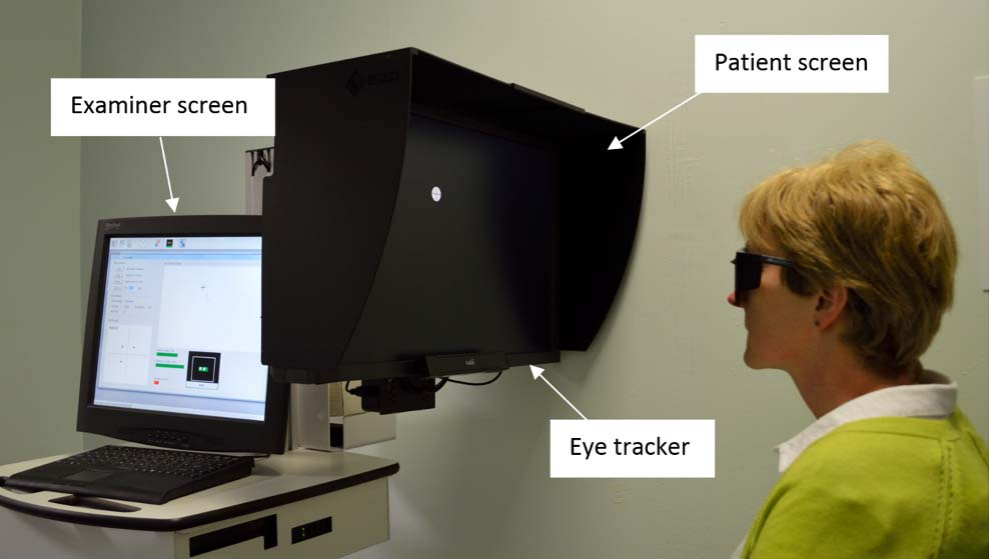
The threshold SVOP instrument showing the patient screen, eye tracker position, and examiner screen.

The suprathreshold version of SVOP has been previously described.^9,10^ Briefly, the SVOP device consists of a personal computer, two display screens (one for the patient and another for the examiner), and an eye tracker. The eye tracker assesses patient eye gaze responses to visual field stimuli presented on the display screen and a software algorithm determines if the stimuli have been perceived based on the direction and amplitude of a subject's eye gaze responses rather than a patient response button. The only task required of the patient is to refixate on a peripheral stimulus, if they have seen it, which then becomes the fixation target for the next peripheral test point. A peripheral stimulus will be presented only if the system determines that the patient is fixating correctly on the fixation target. The eye tracker also provides “real time” data on eye location allowing screen coordinates of visual field stimuli to be calculated based on the position of the patient, meaning that there is no requirement for a chin rest. In other words, the size and position of the stimuli can be adjusted automatically and continually to compensate for changes in the patient's position during testing. Figure 1 shows the SVOP instrumentation in use.

Threshold SVOP uses the same underlying principles as suprathreshold SVOP, with the exception that the fixation target remains in place until a test stimulus is seen and an appropriate eye gaze is made. In the suprathreshold test, the fixation target disappears when a peripheral stimulus is shown so as to incite an eye movement reaction. In a threshold test where many stimuli will not be seen, this approach would lead to lengthy test durations because refixation time would be required for every presented stimulus.

The eye tracker used for threshold SVOP was an IS-1 model (Tobii Technology, Stockholm, Sweden). The patient display screen was a 24″ ColorEdge CG243W LCD display (Eizo Corporation, Hakusan, Japan) controlled by a FirePro 2270 graphics card (Advanced Micro Devices, Sunnyvale, CA) providing native 10-bit color depth support. The display was calibrated to generate accurate representation of the varying stimulus luminance levels required for threshold testing. Briefly, the screen calibration technique uses an accurate Look-Up Table (LUT) pairing the grey-levels of each pixel to the corresponding required luminance levels. This enabled the accurate reproduction of the background and stimulus luminance levels required for threshold testing.^13^ This method of calibration was performed once at the beginning of the study. A second, simpler screen calibration using an x-rite i1 Display Pro Colourimeter (x-rite Inc., Grand Rapids, MI) was used twice during the study to maintain the display color consistency.

The test pattern used was equivalent to the SAP 24-2 test pattern. Peripheral stimuli were presented at size Goldmann III for 200 ms each on a background of 10 cd/m^2^. Stimuli luminance levels replicated the luminance values corresponding to 14-40 dB on SAP. The SVOP threshold sensitivity values were matched in luminance to those of the SAP to allow direct comparison. Thresholds were obtained using a 4-2 bracketing strategy and began by testing four “seed” locations (one in each quadrant), which then were used to set the starting stimulus luminance levels for neighboring locations, which in turn were used to calculate the remaining starting luminance levels.

The room lights were off for SAP and SVOP testing. Participants were seated in front of the patient screen with their eyes aligned with the center of the screen at a testing distance of approximately 55 cm, measured using an eye tracking–based on-screen tool. Monocular testing was made possible using custom made test spectacles allowing transferable, full aperture prescription lenses (55 mm diameter) if required, while also occluding the nontest eye with an infrared bandpass filter which enabled the eye tracker to detect the position of the occluded eye. A calibration sequence, in which the subject was required to follow a stimulus to nine different screen locations, was performed before each test to produce accurate eye gaze data during testing. Before testing commenced, a 20-second demonstration of the test was provided for all subjects. During testing the patient was instructed to follow their natural reaction to fixate towards any peripheral stimulus perceived.

### Modifications to SVOP during the Study

Throughout the study, the testing clinicians (AMcT and LC) liaised with the research engineers (IM, AP, and HB) to suggest improvements. When sufficient improvements were developed, version 1 (v1) of the SVOP software was replaced with version 2 (v2). Key changes between software v1 and v2 were: (1) an indicator for correct patient height and position (before and throughout the test); (2) an improved interactive demonstration test; (3) an increased time period for determining fixation and new fixation target with central pulsating crosshairs to improve fixation; (4) ensuring that starting luminance levels (calculated from “seed” location threshold levels in each quadrant) and subsequent neighboring threshold levels were never below 18 dB; this ensured that threshold point results would never be determined from a single stimulus decision; and (5) two bug fixes (one relating to incorrect screen stimuli positions calculated under rare circumstances, and a second relating to incorrect calculation of the starting luminance level for one field point location).

### SAP Test

SAP was performed using a HFA 750i (Carl Zeiss Meditec). The SITA FAST algorithm was used with the 24-2 test pattern. The eye being tested was aligned to the central fixation point with the aid of the HFA on-screen camera. Monocular testing was performed using an eye patch and, if required, a near prescription was provided using full aperture trial lenses placed in the HFA lens holder. During testing, participants were instructed to fixate on the light in the center of the perimetry bowl throughout the test and to respond to peripheral stimuli by pressing a patient response button. Individuals who were unfamiliar with visual field testing were shown a short demonstration of the test.

## Data Analysis

SVOP tests excluded from analysis were those that were incomplete. SAP tests excluded from analysis were those with a false-positive response rate exceeding 15% (deemed as potentially unreliable as recommended by manufacturer guidelines^14^), and those identified as having eyelid or lens rim artefact. The number of excluded tests is reported.

For group comparison tests, normality assumption was assessed by inspection of histograms and using Shapiro-Wilk tests. For comparison of two groups, Student's *t*-tests were used for normally distributed variables and the Wilcoxon rank-sum test for continuous nonnormal variables. For comparison of more than two groups, 1-way analysis of variance (ANOVA) was used. All tests were 2-sided and a *P* value less than 0.05 was considered statistically significant. Statistical analyses were performed with SPSS version 21 (SPSS Inc., Chicago, IL, USA).

SVOP and SAP tests (excluding repeat tests) were compared by correlating the average sensitivity for each test to produce a Pearson's correlation coefficient (*r*) for each SVOP software version (v1 and v2). This analysis was repeated for each of the 54 visual field test locations. Due to the limitations of the current SVOP LCD screen, it was not possible to generate stimuli with brightness greater than 136.69 cd/m^2^ (corresponding to 14 dB on SAP). Therefore, visual field sensitivity on SAP tests lower than 14 dB were truncated to 14 dB for comparison. To assess the repeatability of SVOP (v1 and v2) and SAP tests, Pearson correlations were calculated for each pair of repeat test point measurements on the same eye (54 points per test).

## Results

### Subjects

A total of 162 subjects took part in the study, including 103 patients with glaucoma (50 female, 53 male) and 59 healthy subjects (40 female, 19 male). Of those with glaucoma, 90 had open angle and 13 primary angle closure glaucoma. Healthy subjects were younger than those with glaucoma (65.7 ± 5.8 vs. 70.6 ± 8.79 years, respectively; *P* < 0.001).

Table 1 shows the number of subjects enrolled to perform SVOP testing with v1 or v2. It also shows the number of SVOP and SAP tests performed (excluding repeat tests), and the number of tests that were excluded from analysis (incomplete SVOP tests and SAP tests deemed unreliable due to false-positive rates greater than 15%, or with artefact). Finally, Table 1 also shows the resultant number of comparison pairs (equivalent SVOP and SAP test results) used for analysis after excluded tests are removed.

**Table 1:**
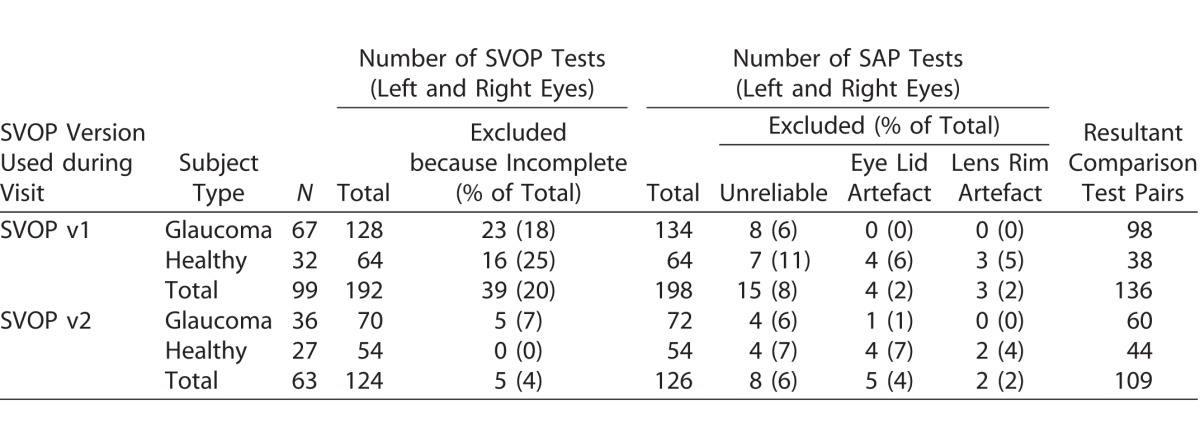
Number of Subjects and Tests (SVOP v1, v2, and SAP) Performed (Excluding Repeat Tests), the Number (and Reason) of Excluded Tests and the Resultant Comparison Test Pairs Used for Analysis after Exclusions

Tables 2 and 3 show the number of subjects who performed repeat testing for SVOP (v1 and v2) and SAP, respectively. They also show the number of tests excluded from analysis and the resultant repeat pairs used for analysis.

**Table 2:**
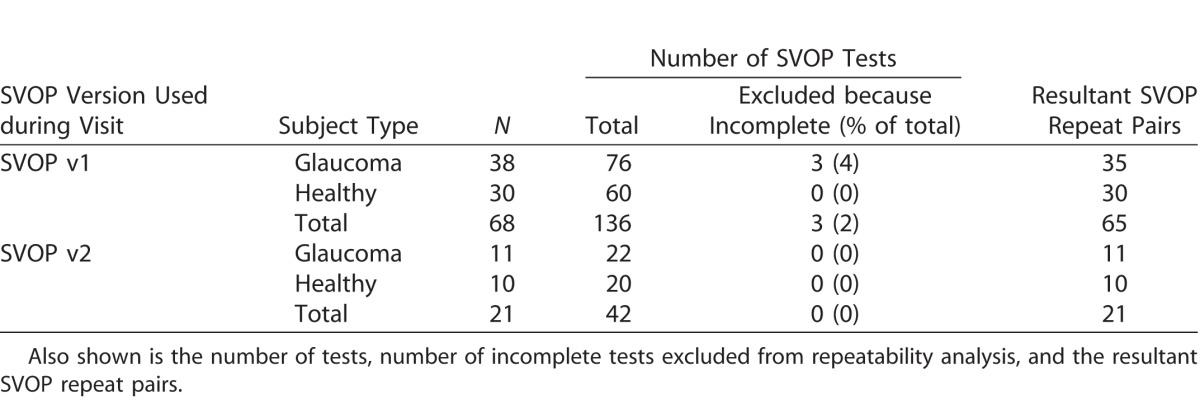
Number of Subjects Who Performed Repeat Testing for SVOP

**Table 3:**
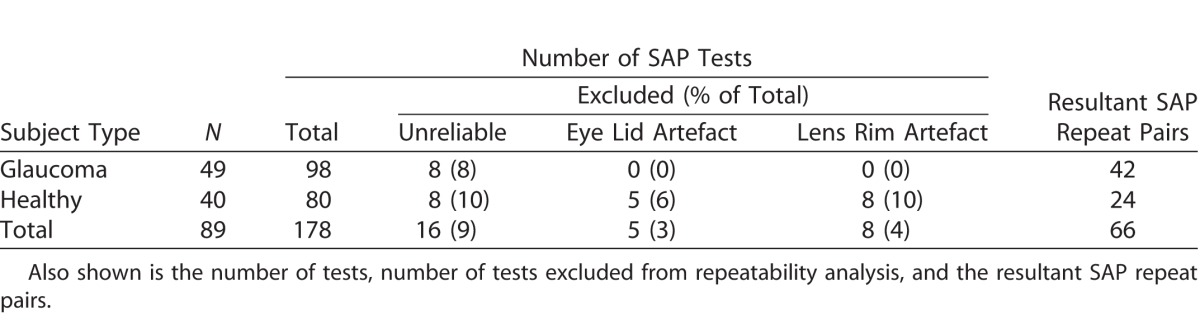
Number of Subjects Who Performed Repeat Testing for SAP

### Comparison of Thresholds Obtained with SVOP and SAP

**Figure 2:**
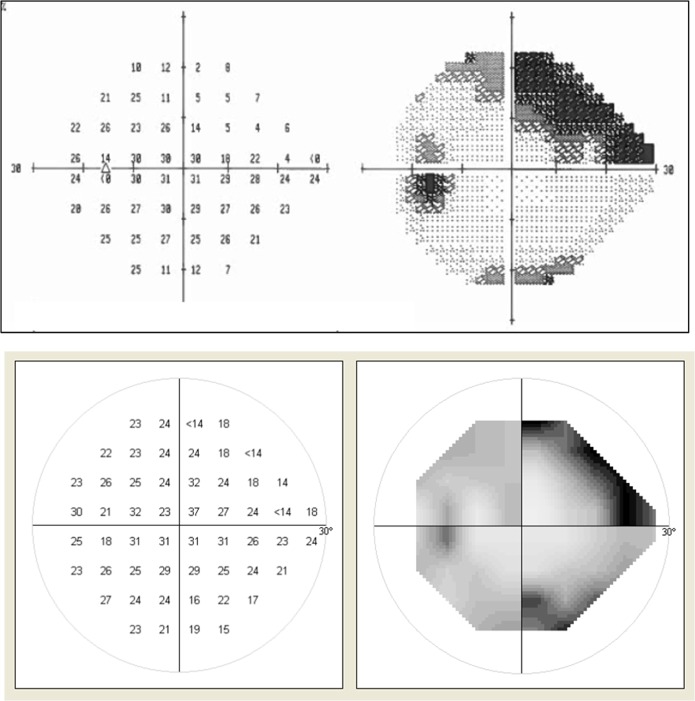
Example test outputs for the left eye of a glaucoma patient. Upper: HFA device. Lower: Threshold SVOP.

**Figure 3:**
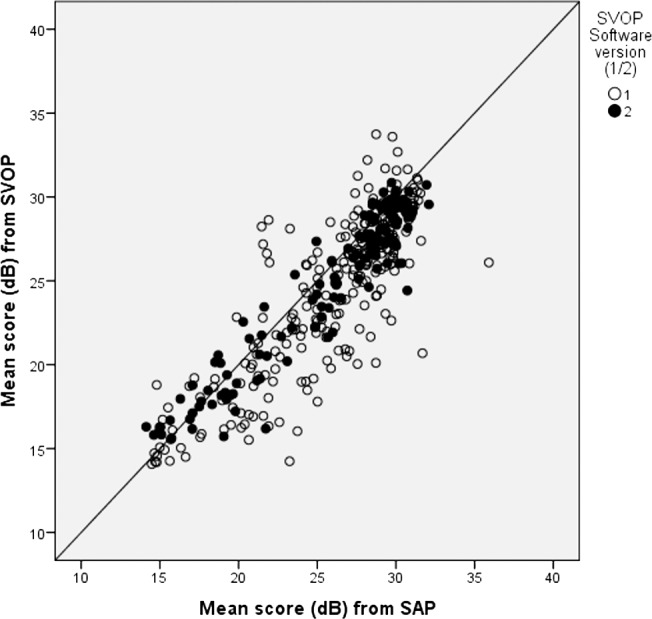
Mean thresholds (dB) for each visual field test. SVOP (v1 and v2) plotted against SAP. For SVOP v1, n = 136, r = 0.83. For SVOP v2, n = 109, r = 0.95.

Overall there was good agreement between threshold sensitivity values obtained with SVOP and SAP. For example, Figure 2 shows the test outputs (SAP and SVOP) for the left eye of a glaucoma patient. Figure 3 shows the mean threshold values for SVOP and SAP for SVOP v1 and v2 (*r* = 0.83 for v1 and 0.95 for v2).

**Figure 4:**
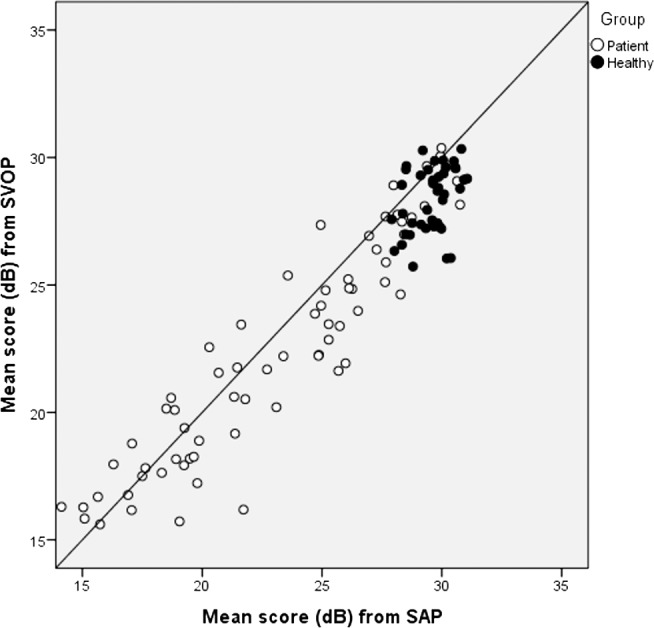
Mean thresholds (dB) for SVOP (v2) plotted against SAP showing patients and healthy subjects (n = 109).

Figure 4 shows the average thresholds for patients and healthy subjects separately for SAP and SVOP v2. Healthy subjects had a tight distribution of points with sensitivity ranging approximately 28 to 31 dB for SAP and 26 to 30 dB for SVOP.

**Figure 5:**
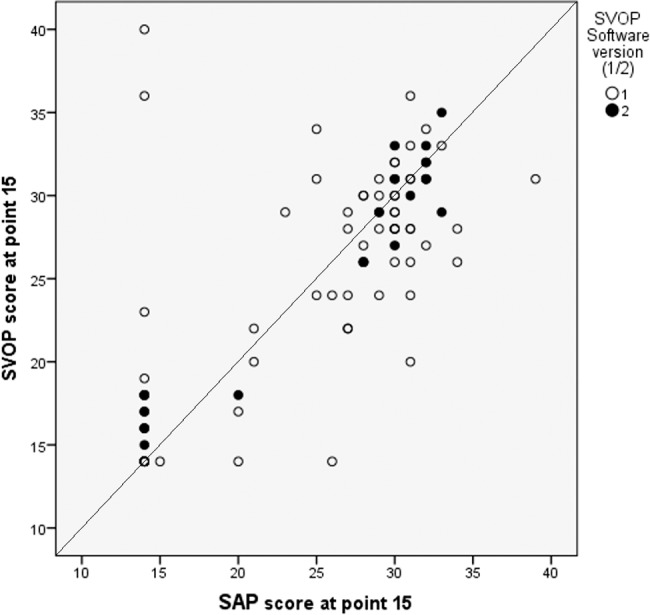
Mean thresholds (dB) for one visual field test location (3° temporal, 9° superior) for SVOP v1 and v2 plotted against SAP. n = 136 for SVOP v1, n = 109 for SVOP v2 (glaucoma patients and healthy controls).

Analysis also was performed on individual visual field locations. This is of interest as the expected thresholds across the visual field are expected to differ dependent on the position tested. For example, Figure 5 shows threshold values for one visual field test location (3° temporal, 9° superior) for SVOP v1 and v2 and includes glaucoma patients and healthy subjects.

**Figure 6:**
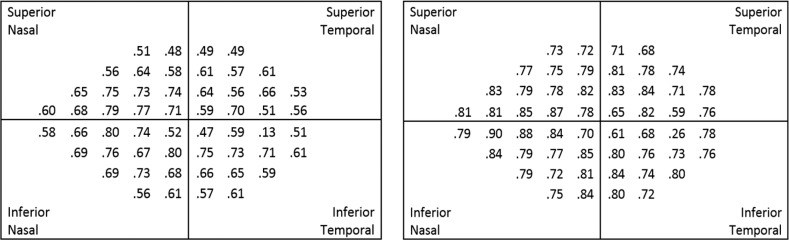
r values for SVOP v1 (left) and v2 (right) compared with SAP, for each individual test point location. n =136 for SVOP v1, n =109 for SVOP v2.

Similar comparisons at each of the 54 visual field point locations were performed. Figure 6 shows the Pearson correlations (*r*) between tests at each location. There was closer agreement between SAP and SVOP v2 than between SAP and SVOP v1.

### Repeatability of Threshold SVOP and SAP

**Figure 7:**
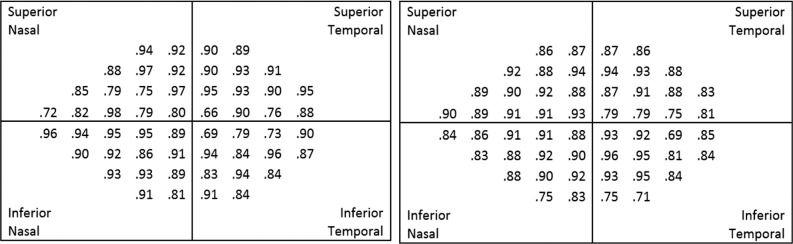
Pearson correlations for repeatability of SVOP v2 (left) and SAP (right), at each visual field test location. n = 21 for SVOP v2, n = 66 for SAP.

To assess the repeatability of the numerical scores, Pearson correlations were calculated for each pair of repeat measurements on the same eye. For SVOP v1, the average correlation over the 54 points was 0.66, with a range of 0.37 to 0.85, indicating only moderate repeatability. The lowest correlation of 0.37 was an outlier, corresponding to the blind spot. For SVOP v2 and SAP, the corresponding figures were 0.88 (0.66–0.98) and 0.87 (0.69–0.96). Thus, the repeatability of SVOP v2 was much better than that for v1, and comparable to SAP. The individual test location repeatability Pearson correlations, for SVOP v2 and SAP, are shown in Figure 7.

### Test Times

The mean test duration for patients performing the SITA Fast test (number of tests [N] = 245, mean [M] = 269.4, standard deviation [SD] = 88.2) was significantly shorter than SVOP v1 (mean difference of 2 minutes 31 seconds, N = 151, M = 420.5, SD = 161.3; *P* < 0.001), and also was significantly shorter than SVOP v2 (mean difference of 4 minutes 9 seconds, N = 76, M = 518.3, SD = 155.2; *P* < 0.001). Additionally, the test duration for patients performing SVOP v2 was significantly longer than SVOP v1 (mean difference of 1 min 38 seconds; *P* < 0.001).

The mean test duration for healthy subjects performing SAP (N = 143, M = 189.1, SD = 34.0) was significantly shorter than SVOP v1 (mean difference of 4 minutes 9 seconds, N = 91, M = 437.6, SD = 157.9; *P* < 0.001) and SAP was significantly shorter than SVOP v2 (mean difference of 5 minutes 31 seconds, N = 64, M = 520.3, SD = 148.5; *P* < 0.001). Additionally, test duration for healthy subjects performing SVOP v2 was significantly longer than SVOP v1 (mean difference of 1 minute 23 seconds; *P* = 0.003).

Finally, the mean SAP test duration for patients (N = 262, M = 268.2, SD = 86.8) compared to healthy subjects (N = 157, M = 192.5, SD = 37.7) was significantly longer (*P* < 0.001).

## Discussion

This study revealed good correlation between sensitivity values obtained using SAP and those obtained using a new eye-tracking perimeter. There was excellent agreement between average visual field sensitivity using SVOP v2 and SAP, demonstrating that SVOP can determine threshold visual field values accurately when compared to the accepted gold standard in patients with glaucoma. The study also showed SVOP to have good repeatability, similar to SAP for SVOP v2. Together these results suggested that threshold SVOP could have use for evaluating glaucomatous visual field loss, particularly in patients who may struggle with conventional white-on-white automated perimetry.

Overall average SVOP v2 and SAP thresholds showed close agreement. A pointwise comparison of SAP and SVOP thresholds revealed agreement between SAP and SVOP varied depending on location, with *r* values, excluding the blind spot, ranging from 0.23 to 0.64 for SVOP v1, improving to 0.46 to 0.82 for SVOP v2. Correlation was lowest in the superior and central test locations. Poorer correlation in the central locations is likely to be due to the difficulties of the eye tracker discriminating the small changes in fixation needed to evaluate the central visual field from eye gaze errors. The eye gaze error reported by the eye tracker manufacturer is <0.5°. However, this can be greater in more uncontrolled conditions, such as during an SVOP test where a patient's head may move or they may be wearing corrective lenses. The result is that some stimuli that were perceived by the subject may not have been recorded as “seen” by SVOP; consequently, this resulted in lower threshold (dB) values being produced by SVOP than SAP in the central 4 test points, which is a finding reported previously by another group who have developed eye gaze perimetry.^15^

The lower correlation for test points in the superior visual field was attributed to these points sometimes being tested towards the end of the test, meaning that their presentation became more predictable due to repetitive testing in these locations. The reason for these test locations being left until the end stages of a test primarily is because less screen area is available for fixation that allow these points to be tested and, hence, they are less likely to be selected during earlier stages of the test. With knowledge of these limitations and understanding of the data generated by the eye tracker these issues can be resolved, for example by changing the fixation requirements depending on the test stimulus eccentricity of a point to be tested, and by increasing the priority of testing certain locations before the end of the test.

Overall SVOP v2 showed good repeatability with a Pearson correlation of 0.88. The repeatability of SVOP v2 on a point-by-point basis ranged from 0.66 to 0.98 (Fig. 7), with 45 of 54 points (83.3%) having Pearson correlations of greater than 0.80. In comparison, repeatability of SAP was 0.87, ranging from 0.69 to 0.96, with 47 of 54 (87.0%) SAP test points with correlations greater than 0.80. The points with poorest repeatability for both tests were those in the temporal field close to the blind spot. This also was the case for SVOP v1, which showed an even greater variability in the blind spot area.

SVOP v2 showed better agreement with SAP and better repeatability compared to SVOP v1. It was felt that improving the participant starting position provided the greatest benefit, but also the small changes and bug fixes ultimately contributed to the overall improvements.

The SVOP threshold test currently has limitations. First, it does not produce stimuli brighter than equivalent to 14 dB on SAP. With a different display screen, this could be improved to 10 dB, but it would not be possible to achieve the brightness equivalent to 0 dB on SAP. Although the inability to generate stimuli with intensity greater than 14 dB is a limitation of SVOP, a recent study by Gardiner et al.^16^ has suggested that due to a reduction in the asymptotic maximum response probability, SAP may be unreliable in test locations with sensitivity below 15 to 19 dB. This would make detection of progression in areas of the visual field with sensitivity below this level problematic and illustrates the need for alternative testing strategies to detect progression in areas of the visual field with advanced loss. The display screen also requires calibration to produce and maintain accurate luminance presentation. The display calibration processes (factory and in-the-field maintenance calibrations) could be automated easily.

A further limitation of threshold SVOP is that some tests were not completed (11% and 5% of initial SVOP tests attempted were incomplete for v1 and v2, respectively). Incomplete tests largely were due to poor quality eye tracking data, meaning that the test could not proceed as it was unable to determine accurately whether the subject was fixating correctly. We observed a larger proportion of tests on glaucoma patients being incomplete compared to healthy subjects (15% and 3%, respectively, for SVOP v1, 7% and 0%, respectively, for v2). This is likely because the eye tracker was developed for a normal population and occurrences of ocular abnormalities (which could interfere with the eye tracking) are more likely to occur in the patient population. The Tobii eye tracker uses video identification of corneal and pupil reflexes. Incomplete tests due to poor eye tracking can occur when the quality of the image of the pupil margin and corneal reflex is impaired (e.g., due to factors, such as dry eye, high diopter lenses, irregular pupil shape, and eye makeup). The rate of incomplete tests would need to be reduced to make the system more acceptable for use in clinics. However, newer models of eye trackers have introduced proprietary developments to improve eye tracking and we have begun work on a further iteration of the technique using newer eye tracking hardware with promising results when retesting some patients who produced incomplete tests in this study.^17^ To further improve SVOP, collaboration with eye tracking manufacturers would be required to better understand the issues and produce an eye tracker designed with the requirements of SVOP in mind. In addition, there also is useful information on eye tracking quality, which can be measured before the test (during the eye tracking calibration) and during the test, which could be used to ascertain how likely it is the test will complete.

It also is important to acknowledge that SVOP test duration was significantly longer than SAP, for both subject groups and for both SVOP software versions. This primarily is due to the way in which the system determines the threshold at each visual field test location. The SITA fast algorithm, which was used for SAP testing, uses a model to estimate threshold values for each point based on responses to stimuli presented at that location, as well as information gathered from nearby locations, and tests considerably fewer points than the SVOP test, which uses the 4-2 bracketing technique. Several techniques can be incorporated into SVOP to improve test time. One simple example would be to reduce the amount of time SVOP waits to make an “unseen” decision. Currently, this is set to 1 second, but a dynamic value based on patient response time for “seen” stimuli could be used. Given that near to 50% of stimuli in a threshold test can be unseen, this would represent considerable improvement in test time. SVOP v2 was on average longer to complete than v1. The reason for this primarily was because the number of stimulus presentations was greater with v2 due to the modification, which ensured that starting luminance levels (calculated from neighboring completed test locations) were never below 18 dB and because of the longer period used to determine fixation. There was no significant difference in the test times between patients and healthy subjects performing SVOP in contrast to SAP, where on average patients had a longer test duration. This simply may be due to the difference in algorithms used to determine threshold. However, there are aspects of SAP testing that SVOP bypasses (e.g., motor response time), which could contribute to this effect and this warrants further investigation.

Our study design also was limited by the need to compare SVOP to SAP. Although SAP is the gold standard, and the obvious choice for device validation, SAP itself is subject to variability. Difficulties also were encountered with 5% of initial SAP tests, where the data were deemed unreliable due to false-positive responses exceeding 15% and subsequently excluded from analysis. In all 30 of the “unreliable” SAP tests the corresponding SVOP test was completed successfully. Given the overall results demonstrating the accuracy of threshold SVOP, this indicates that in these subjects SVOP would have provided a useful result where SAP did not, indicating that threshold SVOP, even in its current form, may prove useful in a group of patients who struggle to perform SAP reliably. False-positive responses on SAP occur if the subject responds by pressing the button when no stimulus is present. It is an indicator that the subject may be “trigger happy” and affects the validity of the visual field results. SVOP requires an eye gaze response in the direction of the test stimulus and, therefore, it is very difficult to make a conscious false-positive response.

Threshold SVOP is an extension of our previously described suprathreshold SVOP,^11^ which itself was an extension of work performed by Bertil Damato in 1985.^18^ As would be expected the testing times for threshold SVOP are longer compared to suprathreshold SVOP (143 seconds longer in adults and 82 seconds longer in healthy adults). Both forms of SVOP testing perform well in healthy subjects and glaucoma patients (suprathreshold SVOP showed a sensitivity and specificity of 69.3% and 96.6% in adults when analyzing individual test points).

In this study, we demonstrated an alternative method of measuring threshold visual fields that is repeatable and compares well with the current gold standard. Although further work is required to achieve a faster test and improve test accuracy in the central locations, we demonstrated how iterative improvements can be made through software. More data are required to create a larger database of normative SVOP thresholds and assess more subtle differences between SVOP and SAP (for example correlation at different retinal sensitivities or stages of glaucoma). Further developments and improvements to the SVOP system are possible through advances in eye tracking technology and newer models of eye tracker currently are available that may improve eye tracking in patients who have proved difficult to test thus far. The second part to this study demonstrates how a further iteration of the threshold SVOP system can improve on the rates of incomplete testing, which, coupled with positive patient feedback, make the test a useful and acceptable form of perimetry.^17^ Additional future work will be necessary to assess other outputs available from threshold SVOP tests, such as saccadic reaction times, which may provide additional diagnostic information,^19^ and correlation of threshold SVOP with retinal nerve fiber layer density.

**Figure 8:**
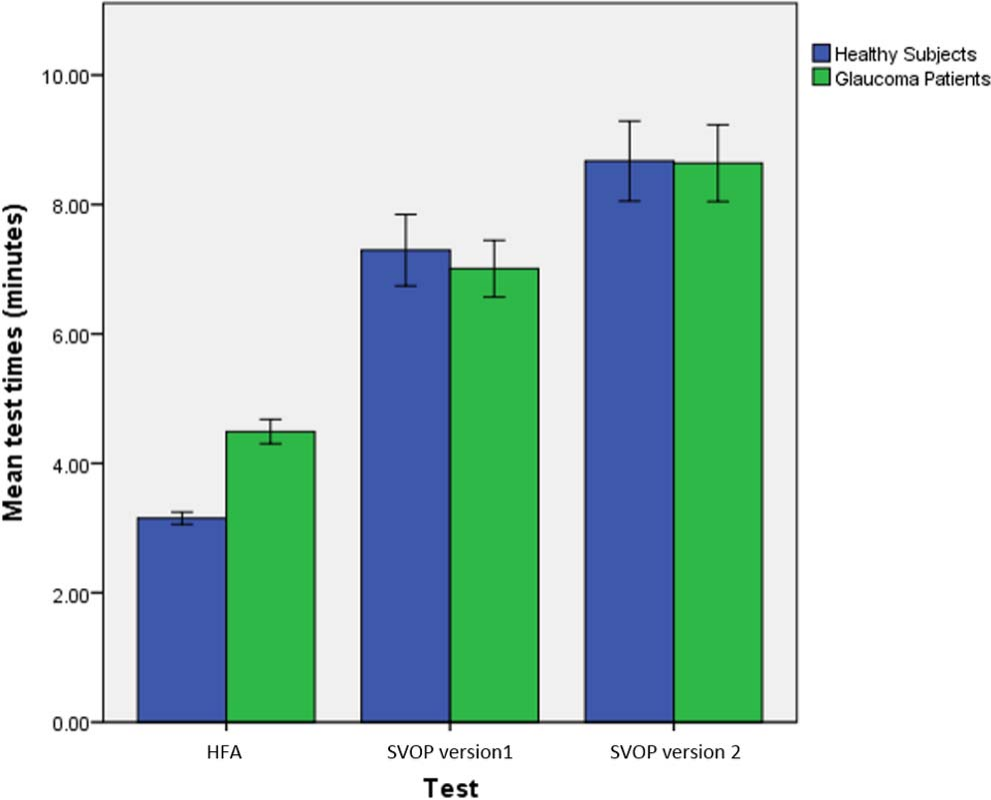
Mean test times for SAP and SVOP tests, for healthy subjects and patients. Error bars: 2 standard errors of the mean.
